# Current status of monkeypox vaccines

**DOI:** 10.1038/s41541-022-00527-4

**Published:** 2022-08-17

**Authors:** Marion F. Gruber

**Affiliations:** International AIDS Vaccine Initiative, 125 Broad Street, 9th Floor, New York, NY 10004 USA

**Keywords:** Drug regulation, Viral infection

Monkeypox disease is caused by infection with the monkeypox virus, an orthopoxvirus belonging to the same poxviridae family as variola and vaccinia viruses. Monkeypox disease was initially diagnosed in 1970 in the Democratic Republic of the Congo (DRC) and has since spread to other countries in Africa, in particular regions of West and Central Africa where it is endemic^1^. Prior to 2022, occasional outbreak outside Africa were linked to international travel or imported animals^2^. In May 2022, multiple clusters of monkeypox were reported in European countries and North America. The number of weekly reported new cases has since dramatically increased resulting in the WHO declaring this outbreak a public health emergency of international concern^3^. On August 4, 2022, monkeypox was declared a public health emergency in the US^4^. As of August 3, 2022, more than 25,000 confirmed cases have been reported to WHO across 85 countries with the majority of the cases reported from the European region and regions of the Americas^5^. Males between 18–44 years of age are disproportionally affected by this outbreak and accounting for 79% of cases. Most of those affected by the current outbreaks are gay, bisexual, or other men who have sex with men. According to the U.S. Center for Disease Control and Prevention (CDC), many of the initial patients reported international travel prior to the onset of symptoms, but since late June 2022, an increasing number of cases are now linked to local community transmission^6^.

The WHO certified the eradication of naturally occurring smallpox in 1980 but because of concerns due to the threat of bioterrorism and monkeypox outbreaks at the start of the 21st Century, additional vaccinia-based smallpox vaccines were licensed. The 1st generation smallpox vaccines such as Dryvax, consisting of the New York City Board of Health (NYCBH) strain of vaccinia, the Lister/Elstree and the Ikeda vaccinia virus strains were lymph derived and grown on the skin of animals. In contrast the 2nd and 3rd generation vaccines were produced using cell culture techniques with the goal to improve their safety profile. In the USA, there are currently two licensed smallpox vaccines. ACAM2000, a replicating vaccinia virus-based 2nd generation smallpox vaccine was licensed in 2007 by the US Food and Drug Administration (FDA) and is derived from a clone of Dryvax. It is indicated for active immunization against smallpox but not monkeypox disease for persons determined to be at high risk for smallpox virus infection^7^. However, ACAM2000 may cause serious adverse reactions, including myopericarditis in smallpox naive individuals, and is thus contraindicated in severely immunocompromised individuals. Alternative further attenuated vaccinia virus-based vaccines were developed using serial passage of the vaccinia virus in primary cell culture or eggs as the method for attenuation. Jynneos, a live, non-replicating attenuated 3rd generation smallpox vaccine was licensed by the FDA in 2019. It is derived from Modified Vaccinia Ankara (MVA) attenuated strain which, in turn, was generated by more than 500 serial passages of the vaccinia virus Ankara strain in chick embryo fibroblast (>570 times). Jynneos is indicated for the prevention of smallpox and monkeypox disease in adults 18 years of age and older determined to be at high risk for smallpox and monkeypox disease^7^. This vaccine was previously approved for protection against smallpox disease for persons 18 years of age and older by the European Medicine Agency (EMA) in 2013, under the trade name IMVANEX, and by the Public Health Agency of Canada (PHAC) in 2013 under the trade name IMVAMUNE. PHAC extended the indication for IMVAMUNE in 2020 to include monkeypox and related orthopoxvirus infection and in July 2022, EMA extended the indication of IMVANEX to include monkeypox and disease caused by vaccinia virus^8,9^. LC16 m8 is a live, replicating attenuated 3rd generation vaccines derived from the Lister (Elstree) strain of vaccinia and licensed since 1975 for active immunization against smallpox in Japan. In August 2022, Japan extended the indication of this vaccine to include protection against monkeypox^10^. Because of their attenuated phenotype, these vaccines have an improved safety profile and can be administered to immunocompromised individuals. The effectiveness of these vaccines against monkeypox is inferred from data from animal studies demonstrating protection against the monkeypox virus in non-human primates vaccinated with these vaccines and clinical trials demonstrating their immunogenicity in human subjects.

On June 14, 2022, the WHO issued interim guidance stating that mass vaccination is not required nor recommended for monkeypox and advocates for judicious use of currently available vaccines as supply is limited^11^. WHO stresses for vaccination programs be backed by surveillance and contact-tracing, and accompanied by a strong public health messaging, robust pharmacovigilance, ideally in the context of collaborative vaccine effectiveness studies with standardized protocols and data collection tools. In this regard, WHO held two consultations of global experts on June 2 & 3rd and August 2, 2022, to identify current knowledge gaps regarding monkeypox transmission, epidemiology, clinical disease characteristics, and country perspectives in terms of opportunities and research priorities. As there is uncertainty about how well the vaccines will work in protecting people in the current outbreak, strategies and study designs to evaluate vaccine effectiveness in protecting against monkeypox, to better understand risk factors and the natural history of monkeypox disease were also discussed^12,13^. WHO recommended for countries to make efforts to use available monkeypox vaccines within a framework of collaborative clinical efficacy and safety evaluations.

In an effort to interrupt and/or reduce human-to-human transmission of monkeypox and thus, curb the spread of monkeypox in at-risk populations, countries in Europe and the American region have started vaccination programs offering the vaccines to individuals with high-risk exposures, e.g., sexual health clinic attendees with Sexually Transmitted Infections and/or within settings where transmission is happening^13,14^. These countries are planning studies in pre-exposure and postexposure settings to evaluate vaccine effectiveness and safety in populations at risk and in special populations such as pregnant women and immunocompromised individuals. Studies will also evaluate single-dose regimen and fractional doses in order to extend currently available vaccine supplies^13^.

There is a significant shortfall of monkeypox vaccines, relative to current anticipated needs, and manufacturing ramp-up will take time to supply even high-income countries with sufficient doses of monkeypox vaccines thus, presenting challenges to countries in combating the fast-growing outbreak of monkeypox. As of August 4, 2022, the US Department of Health and Human Services’ (HHS) Administration for Strategic Preparedness and Response (ASPR) delivered more than 602,000 doses of Jynneos from its Strategic National Stockpile to jurisdictions in the US and announced that an additional 2.5 million doses had been ordered bringing the federal government’s available supply to more than 6.9 million in the coming months^15^.

In an effort to extend the limited vaccine supply, in the US, public health officials are considering dose-sparing approaches, e.g., using one dose of Jynneos administered subcutaneously (s.c.) instead of the licensed two doses and/or using two doses administered at a fraction of the standard dose by the intradermal route (i.d.) of administration of the attenuated MVA vaccine^13^. In these situations, the safety and immunogenicity of the vaccine when administered at doses and routes of administration (RoA) different than licensed will need to be evaluated in the context of appropriately designed clinical studies. This could be achieved by way of conducting immunogenicity studies comparing vaccinia-specific neutralizing antibody titers induced by the vaccine administered at fractional doses and/or administered by different RoA to those induced using currently licensed doses and RoA as well as comparing the local and systemic reactogenicity in subjects enrolled. In the US, FDA can authorize the emergency use of an unapproved medical product or an unapproved use of an approved medical product for certain emergency circumstances under Emergency Use Authorization (EUA)^16^. The latter would require a determination by the Secretary of HHS of a public health emergency as occurred on August 4, 2022^4^. On August 9, 2022, FDA authorized emergency use of JYNNEOS to increase the vaccine supply^17^. This allows health care providers use of a) two doses (0.1 ml each rather than 0.5 ml each) of JYNNEOS 4 weeks apart via the i.d. RoA to individuals 18 years of age and older determined to be at high risk for monkeypox and b) two doses (0. 5 ml each) of Jynneos 4 weeks apart via the s.c. RoA to individuals younger than 18 years determined to be at high risk of monkeypox infection. Alternatively, and different from EUA, individuals could be enrolled into a clinical study under Expanded Access (EA) to administer fractional doses and alternate RoA of the vaccine. EA is a regulatory mechanism with the primary purpose to prevent or treat the patients’ disease, not to obtain safety or effectiveness data of the product provided certain criteria are met^18^. This mechanism was used during the shortage of Yellow fever vaccine^19^.

Monkeypox is now a disease of international relevance and concern and resources must be mobilized to curb this outbreak globally. According to a statement made by the Acting Director of the Africa CDC in a press briefing on July 21, 2022, Africa, where monkeypox disease has been endemic for decades, has no vaccines against monkeypox^20^. The current COVID-19 pandemic that still poses global health challenges has underscored the importance of globally accessible and affordable vaccines to protect the global community against emerging and/or reemerging infectious diseases. Therefore, high-income countries, in addition to their national monkeypox outbreak responses, must continue efforts to combat monkeypox in endemic countries. National and international governments and stakeholders must work together to provide global, equitable access to monkeypox vaccines and to support the international response to the monkeypox outbreak.

## References

[1] Moore, M. J., Rathish, B., & Zahra, F. *Monkeypox StatPearls* (StatPearls Publishing, 2022). 34662033

[2] Bunge, E. M. et al. The changing epidemiology of human monkeypox—a potential threat? A systematic review. *PLoS Neglected Tropical Dis.* **16**, e0010141 (2022). 10.1371/journal.pntd.0010141 PMC8870502 35148313

[3] WHO Director—General declares ongoing monkeypox outbreak a Public health Emergency of International Concern. https://www.who.int/europe/news/item/23-07-2022-who-director-general-declares-the-ongoing-monkeypox-outbreak-a-public-health-event-of-international-concern (2022).

[4] Biden-Harris Administration Bolsters Monkeypox response, HHS Secretary Becerra Declares Public health Emergency. https://www.hhs.gov/about/news/2022/08/04/biden-harris-administration-bolsters-monkeypox-response-hhs-secretary-becerra-declares-public-health-emergency.html (2022).

[5] WHO—Multi-country outbreak of monkeypox External Situation report. https://www.who.int/publications/m/item/multi-country-outbreak-of-monkeypox--external-situation-report--2---25-july-2022 (2022).

[6] Centers for Disease Control and Prevention (CDC). Technical report: Multi-National Monkeypox Outbreak, United States. https://www.cdc.gov/poxvirus/monkeypox/clinicians/technical-report.html (2022).

[7] Food and Drug Administration (FDA) Vaccines licensed for use in the United States. https://www.fda.gov/vaccines-blood-biologics/vaccines/vaccines-licensed-use-united-states

[8] GlobeNewswire. Health Canada Extends Approval of IMVAMUNE to Immunization against Monkeypox and Orthopox viruses. https://www.globenewswire.com/news-release/2020/11/12/2125193/0/en/Health-Canada-Extends-Approval-of-IMVAMUNE-to-Immunization-against-Monkeypox-and-Orthopox-Viruses.html (2022).

[9] European medicines Agency (EMA)—EMA recommends approval of Imvanex for the prevention of monkeypox disease. https://www.ema.europa.eu/en/news/ema-recommends-approval-imvanex-prevention-monkeypox-disease (2022).

[10] Healthworld.com. Japan approves smallpox vaccine to prevent monkeypox. https://health.economictimes.indiatimes.com/news/industry/japan-approves-smallpox-vaccine-to-prevent-monkeypox/93321404 (2002).

[11] WHO. Vaccines and immunization for monkeypox: Interim Guidance. https://www.who.int/news/item/14-06-2022-vaccines-and-immunization-for-monkeypox--interim-guidance--14-june-2022 (2022).

[12] WHO. WHO Monkeypox research. What are the knowledge gaps and priority research questions? https://www.who.int/news-room/events/detail/2022/06/02/default-calendar/who-monkeypox-research--what-are-the-knowledge-gaps-and-priority-research-questions (2022).

[13] WHO. WHO Monkeypox research. What study designs can be used to address the remaining knowledge gaps for monkeypox vaccines? https://www.who.int/news-room/events/detail/2022/08/02/default-calendar/who-monkeypox-research---what-study-designs-can-be-used-to-address-the-remaining-knowledge-gaps-for-monkeypox-vaccines (2022).

[14] Centers for Disease Control and prevention (CDC). Considerations for Monkeypox Vaccination. https://www.cdc.gov/poxvirus/monkeypox/considerations-for-monkeypox-vaccination.html (2022).

[15] HHS.gov ASPR Administration for Strategic Preparedness and Response. JYNNEOS Monkeypox Vaccine Distribution by Jurisdiction. https://aspr.hhs.gov/SNS/Pages/JYNNEOS-Distribution.aspx (2022).

[16] US Food and Drug Administration (FDA). Emergency use Authorization for Vaccines Explained. https://www.fda.gov/vaccines-blood-biologics/vaccines/emergency-use-authorization-vaccines-explained (2020).

[17] Monkeypox Update: FDA authorizes Emergency Use of JYNNEOS Vaccine to Increase Vaccine Supply. https://www.fda.gov/news-events/press-announcements/monkeypox-update-fda-authorizes-emergency-use-jynneos-vaccine-increase-vaccine-supply (2022).

[18] US Food and Drug Administration (FDA). Expanded Access—Information for Industry. https://www.fda.gov/news-events/expanded-access/expanded-access-information-industry#ExpandedAccess (2019).

[19] Gersman, M. D. et al. Addressing a yellow fever vaccine shortage. *MMWR Morb. Mortal. Wkly. Rep.* **66**, 457–459 (2017). 10.15585/mmwr.mm6617e2 PMC5687078 28472025

[20] Bloomberg US edition. Africa, where Monkeypox is Endemic, gets no vaccines. https://www.bloomberg.com/news/articles/2022-07-28/africa-falling-behind-on-monkeypox-vaccines-as-virus-spreads (2022).

